# Exploring trial acceptability, recruitment, and retention in a pilot optimisation randomised controlled trial of a behavioural intervention to support medication adherence in women with breast cancer: A qualitative process evaluation

**DOI:** 10.3310/nihropenres.14321.1

**Published:** 2026-07-05

**Authors:** Jennifer F Ingram, Elizabeth Travis, Louise H Hall, Erin Raine, Rachel Ellison, Emma McNaught, Hollie Wilkes, Pei Loo Ow, Christopher D Graham, David P French, Daniel Howdon, Jane Clark, Jo Waller, Jacqueline Buxton, Sally J L Moore, Catherine Parbutt, Galina Velikova, Amanda J Farrin, Michelle Collinson, Samuel G Smith*, Sophie M C Green*

**Affiliations:** 1 Leeds Institute of Health Sciences, University of Leeds, Leeds, England, LS2 9NL, UK; 2 York Trials Unit, University of York, York, YO10 5DD, UK; 3 Clinical Trials Research Unit, Leeds Institute of Clinical Trials Research, University of Leeds, Leeds, England, LS2 9NL, UK; 4 Department of Psychological Sciences and Health, University of Strathclyde, Glasgow, Scotland, G1 1QE, UK; 5 Division of Psychology and Mental Health, University of Manchester, Manchester, England, M13 9PL, UK; 6 Department of Clinical and Health Psychology, Leeds Teaching Hospitals NHS Trust, Leeds, England, LS9 7TF, UK; 7 Wolfson Institute of Population Health, Queen Mary University of London, London, England, E13 8SP, UK; 8 Independent, Leeds, UK; 9 Medicines Management and Pharmacy Services, Leeds Teaching Hospitals NHS Trust, Leeds, England, LS9 7TF, UK; 10 Leeds Institute of Medical Research at St James's, University of Leeds, St James's University Hospital, Leeds, England, LS9 7TF, UK; 11 Leeds Cancer Centre, Leeds Teaching Hospitals NHS Trust, St James's University Hospital, Leeds, England, LS9 7TF, UK; 12 Edinburgh Clinical Trials Unit, Usher Institute, University of Edinburgh, Edinburgh, Scotland, EH16 4UX, UK

**Keywords:** trial experience, acceptability, recruitment, retention, optimisation trial, factorial trial

## Abstract

**Background:**

Complex factorial trials are increasingly employed to optimise complex behavioural interventions. However, acceptability of such designs to participants are unknown. In this qualitative process evaluation of a pilot optimisation randomised controlled trial (O-RCT) for a behavioural intervention to support medication adherence in women with breast cancer, we explored acceptability of trial participation, alongside barriers and facilitators to recruitment and retention.

**Methods:**

We conducted a 2 ^4–1^ fractional factorial pilot O-RCT evaluating the feasibility of four intervention components aiming to support adherence to adjuvant endocrine therapy in women with breast cancer. 52 women were randomised to one of eight conditions, comprising unique intervention component combinations. Four-months post-randomisation, 20 participants completed a semi-structured interview guided by the Theoretical Framework of Acceptability (TFA) and Theoretical Domains Framework (TDF). Data were analysed using rapid qualitative analysis and findings were mapped onto TFA and TDF constructs.

**Results:**

Generally, participation in the trial was perceived positively ( *affective attitude*). Most women reported easy participation ( *burden*), confidence in capabilities ( *self-efficacy, beliefs about capabilities*), minimal opportunity costs ( *opportunity cost*), and personal benefit from participation ( *perceived effectiveness, knowledge, beliefs about consequences*). Randomisation process understanding ( *coherence*), and questionnaire retention experiences were mixed; women reported reduced motivation to complete questionnaires due to length, repetitiveness, and emotional impact ( *motivation and goals, memory, attention, and decision processes*) *.* Participation decisions were shaped by contextual factors, ( *environmental context and resources*), altruism, personal benefit, and wanting to advance science ( *social/professional role and identity, motivation and goals*).

**Conclusions:**

This qualitative process evaluation suggests participation in an O-RCT was acceptable. Low randomisation coherence and questionnaire burden are notable areas for improvement. Future studies within trials should explore approaches to explaining factorial trials to participants. Barriers and facilitators to recruitment and retention were similar to parallel group randomised trials, suggesting interventions addressing these may be applicable regardless of trial design.

## Plain Language summary

**Background:**

Hormone therapy is commonly prescribed to women with breast cancer for between five and ten years, but many women find it hard to take it as recommended. To help, we developed four types of support: text messages, an information leaflet, a website to help manage side-effects, and a therapy program. In a small trial, 52 women with breast cancer who had been prescribed hormone therapy, were given a different combination of these types of support. The types of support they received was decided by chance. This trial used a new, more complex design that has not been widely used in this setting. Therefore, this study had two aims, to understand:

1. How acceptable it was to take part in the trial
2. What factors may have affected women’s decisions to sign up (recruitment), and to continue in the trial for four months (retention).

**Method:**

We interviewed 20 women who had taken part in the trial. We asked them questions about how acceptable it was to take part in the trial, what motivated them to sign up, and if there was anything preventing them (barriers) from completing the trial.

**Results:**

Overall, participants viewed their trial experience as acceptable. However, not everyone fully understood what it meant to be assigned to a group by chance, and some women found the questionnaires to be too long, repetitive, and emotionally difficult, which made them less motivated to finish them. Many women said they joined the trial to help themselves, to support other women, and to contribute to science.

**Conclusion:**

Overall, this study suggested that taking part in a trial with a complex design did not impact acceptability to take part or deter women from participating. Most reasons for signing up and continuing in the study were similar to trials using simpler designs.

## Introduction

Optimisation randomised controlled trials (O-RCTs) are increasingly used for refining complex interventions, prior to a definitive randomised controlled trial (RCT). ^1^ The Multiphase Optimisation Strategy (MOST) is an engineering-inspired framework for optimising multicomponent behavioural interventions. ^2^ MOST comprises three phases: preparation, optimisation and evaluation. The preparation and evaluation phases are similar to the traditional approach to complex intervention development and evaluation, in which intervention components are developed and pilot tested (preparation phase) and then evaluated as a package, typically using a parallel-group RCT (evaluation phase). ^2^ During the optimisation phase of MOST, fully powered, efficient experimental designs (e.g. factorial designs) are used to estimate main and interaction effects of individual intervention components. Empirical data from the O-RCT can be used to construct an optimal intervention package that balances effectiveness with constraints such as affordability, scalability, and efficiency. ^2, 3^ Despite their increasing use, the acceptability of O-RCT designs to trial participants remains unexplored.

Acceptability is a multifaceted construct reflecting the extent to which people regard an intervention as appropriate, based on their cognitive and emotional responses. ^4^ The Theoretical Framework of Acceptability (TFA) provides a comprehensive, structured approach to measuring acceptability, conceptualising it through seven dimensions: affective attitude, burden, ethicality, intervention coherence, opportunity costs, perceived effectiveness, and self-efficacy. ^5^ However, the most widely accepted definition of acceptability focuses on interventions rather than broader trial participation experience, and there is currently no formal definition of participant experience in research trial contexts. ^4, 6^ Process evaluations in RCTs tend to focus on intervention evaluation (71%) and rarely focus on acceptability of trial participation (15%). ^7^ This is noteworthy given that Medical Research Council guidance emphasises the importance of process evaluations to understand feasibility and acceptability of interventions and their associated trial procedures. ^8, 9^

Acceptability of trial design and conduct could directly impact trial recruitment and retention rates, which are common issues in RCTs. ^10^ In a review of 388 National Institute of Health Research (NIHR) funded RCTs, only 63% achieved recruitment targets, with an average retention rate of 88% (IQR 80–97%). ^11^ Poor retention poses a particular risk in factorial O-RCTs and may threaten the integrity of the trial. In a 2 ^k^ factorial trial design, participants are randomised to experimental conditions made up of different combinations of *k* intervention components. When there is a balance of participants across experimental conditions and effect coding (−1, +1) is used, the factorial design allows estimation of all main and interaction effects. The overall number of participants must be sufficiently large to achieve statistical power, but the number of participants randomised to and spread across each experimental condition in factorial O-RCT’s can be relatively small. ^2^ As such, retention is crucial in the context of O-RCTs, to ensure there are no conditions with zero participants (known as empty cells), which would increase complexity in analysis and interpretation. Assessing acceptability of trial experience in the pilot trial phase provides an opportunity to amend trial procedures, which could improve trial experience, recruitment and retention ahead of a fully powered O-RCT. ^12, 13^

### The ROSETA programme

The ‘Refining and Optimising a behavioural intervention to Support Endocrine Therapy Adherence’ (ROSETA) programme aims to develop and optimise an intervention package to support adherence to adjuvant endocrine therapy (AET) in women with breast cancer. Women with early-stage (I to III) hormone-receptor-positive breast cancer are prescribed AET for 5–10 years to reduce recurrence and mortality. ^14, 15^ However, up to three-quarters of women are non-adherent to AET, ^16, 17^ reducing quality-adjusted life years and increasing recurrence risk. ^18– 20^ Existing interventions have not targeted the full range of barriers to AET adherence. ^21^

**Figure 1.**
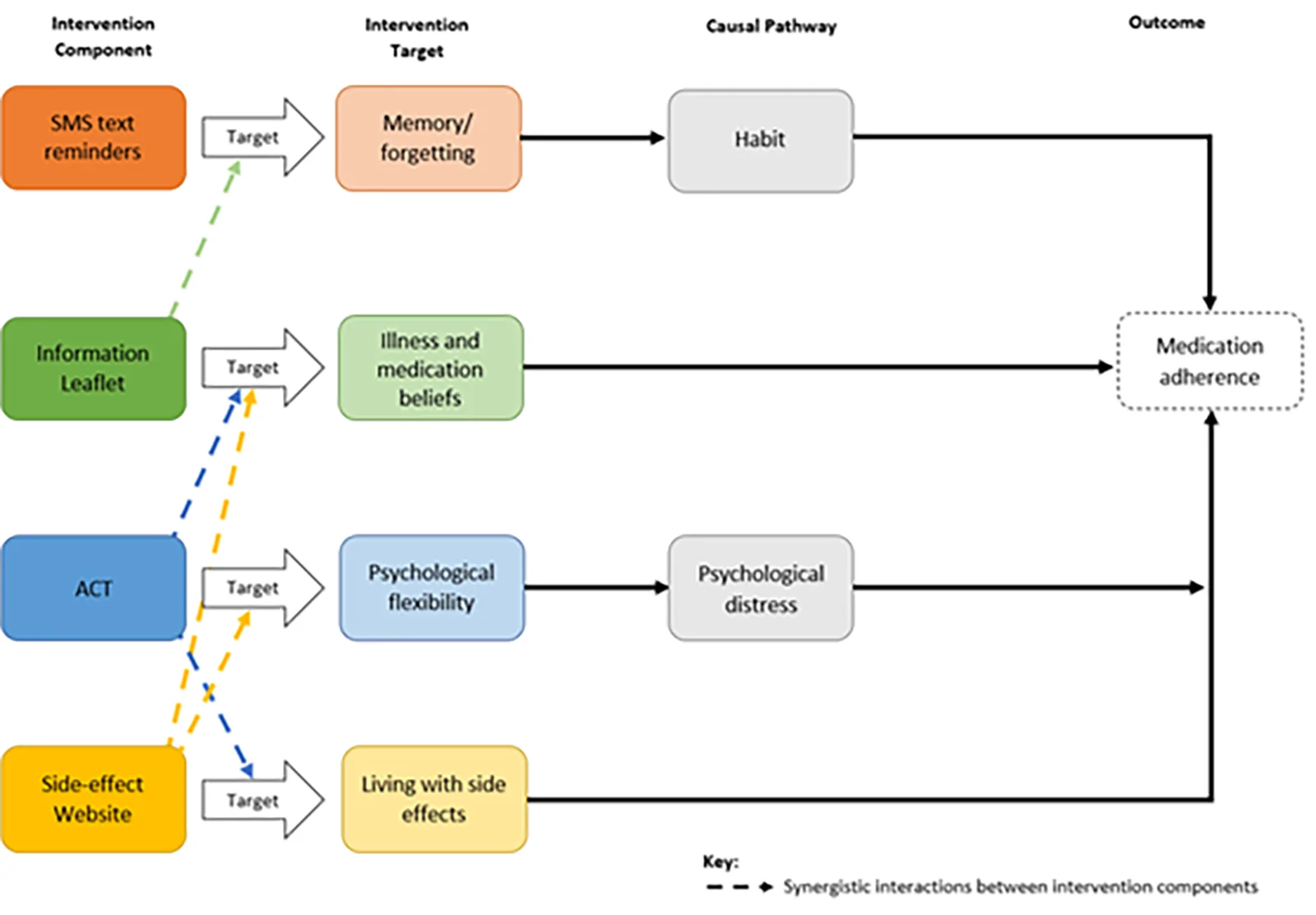
Conceptual model of ROSETA intervention. Figure taken, with permission, from Green et al. ^24^

As part of the preparation phase of MOST, we conducted an exploratory, multi-centre pilot O-RCT using a 2 ^4–1^ fractional factorial design (ISRCTN: 10487576). ^22, 23^ We tested the feasibility of delivering four intervention components: (1) SMS messages targeting forgetfulness, (2) information leaflet addressing concerns and beliefs about AET, (3) acceptance and commitment therapy (ACT)-based guided self-help for increasing psychological flexibility and reducing psychological distress, and (4) a self-management website supporting AET side-effect management ^24^ ( Figure 1).

Participants were randomised to one of eight experimental conditions, each receiving a unique combination of the four intervention components alongside usual care ( Table 1). Across five UK hospital sites, fifty-two adult women with stage I-IIIa breast cancer receiving AET were randomised. Questionnaire-based follow-up was at 2- and 4-months post—randomisation, with each taking approximately 30–45 minutes to complete. Progression to a fully powered O-RCT was deemed appropriate according to progression criteria based on consent rates, adherence to intervention components and availability of the primary outcome data. ^23^ Detailed methodology and pilot trial results are reported elsewhere. ^22, 23^

**Table 1.** 2 ^4−1^ Fractional factorial design used within the ROSETA pilot trial.

Condition	Usual care	SMS	Information leaflet (IL)	ACT	Website (Web)
**1**	Yes	Yes	Yes	Yes	Yes
**2**	Yes	Yes	Yes	No	No
**3**	Yes	Yes	No	Yes	No
**4**	Yes	Yes	No	No	Yes
**5**	Yes	No	Yes	Yes	No
**6**	Yes	No	Yes	No	Yes
**7**	Yes	No	No	Yes	Yes
**8**	Yes	No	No	No	No

Adapted, with permission, from Smith et al. (2025). ^23^

As part of the ROSETA pilot trial process evaluation, we established that all intervention components could be delivered with adequate fidelity and were deemed acceptable to women with breast cancer taking AET. ^25, 26^ Here, we report on the third aim of the ROSETA pilot trial process evaluation, which was to i) explore the acceptability of participant experience in a factorial trial, and ii) explore the barriers and facilitators to recruitment and retention in a factorial trial.

## Methods

The ROSETA pilot trial was approved by Wales Research Authority Research Ethics Committee 3 (21/WA/0322). The pilot trial was prospectively registered with the International Standard Randomised Controlled Trial Number (ISRCTN10487576). Protocols for the ROSETA pilot O-RCT ^22^ and process evaluation ^27^ have been published elsewhere.

### Design

This qualitative process evaluation was nested in the ROSETA pilot O-RCT (ISRCTN: 10487576). We used qualitative semi-structured interviews 4-months post-randomisation to assess participants’ trial experience, guided by the theoretical framework of acceptability (TFA) and the theoretical domains framework (TDF). ^5, 28^ A copy of the interview guide is available in the extended data. ^29^ The TFA was originally designed to evaluate how acceptable a health care intervention is. ^5^ We adapted six constructs of the TFA to assess acceptability of trial participation ( Table 2).

**Table 2.** Theoretical framework of acceptability definitions used.

TFA domain name	Definition used
Affective Attitude	How an individual feels about participating in the trial
Coherence	The extent to which the participants understand the trial processes
Burden	The perceived amount of effort required to participate in the trial
Opportunity Cost	The extent to which benefits, profits, or values must be given to engage in the trial
Perceived Effectiveness	The extent to which the components together are perceived as likely to improve AET adherence
Self-Efficacy	The participant’s confidence in their ability to do everything required in the trial

The TDF evaluates healthcare barriers and facilitators to behaviour change across 14 domains. ^28^ We selected seven TDF domains deemed appropriate to explore participants’ trial experience and perceived barriers to recruitment and retention ( Table 3). This decision was pragmatic to limit participant burden, as the interviews also included questions about acceptability of intervention components and fidelity, related to the wider aims of the process evaluation. ^25– 27^

**Table 3.** Theoretical domains framework definitions used.

TDF domain name	Definition used
Knowledge	Understanding how the trial might impact healthcare
Beliefs about Consequences	Perceived outcomes experienced from trial participation
Beliefs about Capabilities	Perceptions of how easy or difficult it was to participate in the trial
Environmental Context and Resources	Work or home circumstances influencing trial participation
Social/Professional Role and Identity	Understanding their role within the trial as a participant
Motivation and Goals	Motivations to participate in the trial and return questionnaires
Memory, Attention, and Decision Processes	Factors influencing questionnaire completion

### Participants and recruitment

We recruited a subset of participants from the ROSETA pilot O-RCT. Key eligibility criteria for the pilot trial were women aged 18 years or older, who had completed hospital-based treatment for early stage (I to IIIa) breast cancer within the past 12 months, and were prescribed AET (tamoxifen, raloxifene, anastrozole, letrozole, or exemestane). Recruitment took place across five UK NHS hospitals. Full eligibility criteria and recruitment methods are detailed in the protocol. ^22^

All trial participants who agreed to be contacted regarding an optional interview were contacted via email at approximately 3-months post-randomisation, with the view to conducting the interview 4-months post-randomisation. Non-respondents received a follow-up email or phone call after one week. Participants provided either written or verbal (telephone) consent prior to the interview. Telephone consent was provided as an option to reduce barriers to taking part. To obtain telephone consent, a researcher (SMCG) used a telephone script to read the consent statements and initial them on behalf of the patient. A copy of the completed consent form was then emailed to the participant. All consenting participants were invited to interview. The research team (SMCG, LHH, SGS) determined that data were sufficiently “information-rich” to provide a reasonable level of information power for analysis. ^30^

### Data collection

Interviews were conducted by Microsoft Teams or telephone, by a member of the research team (SMCG) between December 2022 and April 2023. The interview schedule served as a flexible guide, and follow-up questions were asked where relevant. All interviews were recorded using in-built recording software, or an encrypted Dictaphone.

### Patient and public involvement

A user advisory group, comprising four women with experience of taking AET, contributed to the development of the interview schedule.

### Analysis

We used rapid qualitative analysis due to limited resources available to undertake this process evaluation. ^31^ The TFA and TDF frameworks informed a deductive analytic approach. One author (JFI) analysed each interview transcript and audio recording using a rapid assessment procedure (RAP) sheet for each participant (see extended data for a blank copy ^29^). ^31– 33^ The RAP sheet comprised three-columns; (1) TFA and TDF constructs, (2) summary notes relating to each construct, and (3) illustrative quotes. Quotes were extracted directly from Microsoft Teams transcripts or were selectively transcribed from telephone interviews. The individual RAP sheets were collated into a single higher-level RAP sheet that synthesised participants’ experiences of the trial. Members of the research team (SMCG, JFI, ET) discussed key findings of the higher-level RAP sheet to enhance reflexivity and include multiple perspectives in interpretation. Key findings from the higher-level RAP sheets were summarised in line with TFA and TDF domains for trial experience, recruitment and retention. Where data reflected conceptually overlapping constructs across the two frameworks, domains were combined in the results to enhance clarity and prevent duplication. The condition the participant was allocated to, the corresponding intervention components they received, and the participant’s age bracket are indicated in brackets following each quote.

## Results

A total of 141 eligible women were approached for participation in the ROSETA trial. Of these, 54 (38.3%) provided consent and 52 were subsequently randomised (36.9%). Follow-up questionnaire completion was 82.7% (43/52) at 2-months and 78.8% (41/52) at 4-months post-randomisation, with no empty cells. Forty-six (88.5%) participants consented to be contacted for interview. Subsequently, five withdrew from the trial, leaving 41 eligible participants who were contacted. Of these, 20 (48.8%) were interviewed, six declined (14.6%) and 15 (36.6%) did not respond. Interviews were conducted between 0 and 46 days following the 4-month follow-up questionnaire being sent. The interviews lasted for an average of 41 minutes (range: 11 to 62 minutes). The mean age of interviewed participants was 57.7 years (SD = 8.34). Additional demographic characteristics are presented in Table 4.

**Table 4.** Participant demographics.

	Overall, n = 52	Interview sample, n = 20
**Age,** mean (SD)	55.2 (10.8)	57.7 (8.34)
**Marital status, ** n (%)		
Married	32 (61.5)	16 (80.0)
Single	6 (11.5)	2 (10.0)
Living with a partner	5 (9.6)	1 (5.0)
Divorced or separated	7 (13.5)	1 (5.0)
Widowed	2 (3.8)	0 (0.0)
**Employment status, ** n (%)		
Full time	22 (42.3)	9 (45.0)
Part time	9 (17.3)	4 (20.0)
Not currently working	9 (17.3)	1 (5.0)
Other	12 (23.1)	6 (30.0)
**Education,** n (%)		
Postgraduate qualification	7 (13.5)	3 (15.0)
Degree level education	10 (19.2)	6 (30.0)
Higher educational qualifications (below degree)	12 (23.1)	6 (30.0)
Vocational Qualifications (NVQ1 + 2)	6 (11.5)	0 (0.0)
A-Level or equivalent	5 (9.6)	1 (5.0)
GCSE/O-Level/CSE	11 (21.2)	4 (20.0)
No formal Qualifications	1 (1.9)	0 (0.0)
**Ethnicity,** n (%)		
White British	43 (82.7)	19 (95.0)
White Irish	1 (1.9)	1 (5.0)
Any other ethnic background	8 (15.4)	0 (0.0)

Adapted with permission from Green et al., (2024). ^25^

### Trial experience

**TFA – Affective Attitude**

Overall, participants reported a positive trial experience. They highlighted the quality of trial communication, availability of remote access, and clear information regarding the withdrawal process.

*“All the information was there, and there was the opportunity … at any point through it I knew … I could of gone this isn’t for me I don’t want to do it anymore.”* (>50 and < 70, C3[SMS, ACT]).

*“It was a positive experience … I think the amount of participation required was … acceptable, you know it didn’t feel like it was a big ask.”* (≤50, C3[SMS, ACT]).

*“The technology side of it is massive.”* (≤50, C4[SMS, Web]).

A few participants were disappointed by their condition allocation in the trial.

*“I was quite disappointed … I went along with it because I’d signed up for it cause I think it’s important that these research studies are done … I was particularly keen on the other one [ACT] for me, would’ve been a more worthwhile study.”* (>50 and < 70, C2[SMS, IL]).

*“I had wished I’d get picked … to do … maybe the psychology side or the side-effects of the drugs and the impact they have on you when … you’re starting on the certain medication that’s required after your diagnosis and treatment.”* (>50 and < 70, C8[no components]).

**TFA – Coherence**

There was mixed understanding about how participants were allocated to intervention components. Some participants demonstrated an understanding of the randomisation process used to allocate them to an experimental condition.

*“I assume you just put people in a big bucket and then draw them out a bit like bingo balls…. So far as I understand its purely arbitrary.”* (>50 and < 70, C2[SMS, IL]).

However, many participants expressed uncertainty regarding the randomisation process. Alternative explanations for their allocation were reported, including beliefs that assignment was based on personal characteristics, questionnaire responses, or stage of cancer.

*“When I found out I was assigned the clinical psychologist, I realised that you had looked at everything and … that that would be the correct person for me.”* (>50 and < 70, C7[ACT, Web]).

*“I kind of guessed that what support you receive … depended on how you answered the first questionnaire … but I don’t know if that was right or not.”* (≥70, C2[SMS, IL]).

*“So, my understanding it was very clear pointed out on that letter. You know, you may get this, this, this, this or this, and I would have thought mine was due to the type [of breast cancer] I had and the severity.”* (≤50, C2[SMS, IL]).

A small number of participants reported having no understanding of the randomisation process.

*“Actually, I don’t think I have got an understanding of how they allocated to different groups to be honest.”* (≤50, C8[no components]).

Two participants also expressed that they were unsure about what the overall purpose of the research was.

*“I mean, obviously I don’t know what you’re doing with your research … that’s up to you.”* (≤50, C2[SMS, IL]).

**TFA – Burden**

Several participants reported that participation in the trial was easy. For example, one participant referred to the intervention summary sheet they were provided with, which summarised what intervention components they would receive at what stages throughout the trial:

“ *Easy [to take part] … I think you know the email popping up…making it clear that the timelines and that kind of thing…if I remember rightly you opt for either email or text reminders, so yeah, having the reminders there*” (>50 and < 70, C6[IL, Web]).

Ease of participation was often attributed to personal circumstances, such as being retired and not having young children, as well as minimal disruption to daily routines, and the availability of online access.

*“I’ve been back at work while I’ve been doing this, and it hasn’t sort of interrupted with work … and a lot of it as I say is either done over the phone or done by email, so it didn’t really interfere on a, you know, a daily basis.”* (>50 and < 70, C3[SMS, ACT]).

*“Easy [to take part], because I suppose in my position [retired] I’ve had the time to think about things, not had to rush off here there and everywhere and catch a bus or whatever.”* (≥70, C2[SMS, IL]).

**TFA – Opportunity Cost**

Most participants did not report any opportunity cost due to participating in the trial. The only opportunity cost, mentioned by a minority of participants, was the time taken to participate in the trial.

*“It costs an investment of your time, but that’s the worthwhile thing to do. But you then have to make that space to do it, but that’s … definitely worth it in my eyes.”* (>50 and < 70, C5[IL, ACT]).

**TFA – Perceived Effectiveness/TDF – Knowledge/TDF – Beliefs about Consequences**

Some participants noted positive personal consequences from their involvement in the trial.

*“‘I’m really glad I did it [trial] … I’ve really benefited from it … I feel it was really good for the way that I like to work things through, so I like reading stuff. I like one to one. I like simple facts and information.”* (>50 and < 70, C1[SMS, IL, ACT, Web]).

*“There’s a bit of a sense of achievement when you’ve completed it.”* (>50 and < 70, C6[IL, Web]).

Several participants also believed the research would have a positive impact for women prescribed AET more generally.

*“I think it’s good to do all this research…because you can’t sort of move on, can you and implement the correct tools for ladies with … the after effects … unless you’ve done some research and got actual proper feedback from people who’ve actually been through it…this felt more tailored towards women who are going through exactly what I’ve been going through.”* (>50 and < 70, C3[SMS, ACT]).

**TFA – Self-Efficacy/TDF – Beliefs about Capabilities**

Most participants felt confident in their abilities to do everything required to participate in the trial, even with some initial uncertainties:

*“When I first started I was really quite worried about it because I thought oh my gosh is it going to be really complicated or hard to do, is it going to be this that or the other, but actually once it got started and I just…know that I got the part with the clinical psychologist and it’s been the best thing ever for me, it’s made me feel so much better.”* (>50 and < 70, C5[IL, ACT]).

**Recruitment:**

**TDF – Environmental Context and Resources**

Several participants described contextual factors that influenced their decision to take part in the trial, such as having an academic or medical background and recognising the value of research, or a family history of cancer that motivated their participation.

*“I am a retired nurse, so I understand, you know, the things … I get the research, I get how important it is.”* (≥70, C4[SMS, Web]).

*“Family history mainly…my mam had breast cancer and my sister, and now me. So, it was mainly to see the more people learn about it or any help that could be out there to stop future generations.”* (>50 and < 70, C8[no components]).

**TDF – Social/Professional Role and Identity/TDF – Motivation and Goals**

Most participants felt their perceived role in the trial and motivation to participate was influenced by wanting to help themselves, support others, and advance research.

*“My motivation was twofold. I think one, selfishly, to get some help if it was available because I could see I was struggling. But two…this second part I think actually was almost more important to me than help for myself, is to get help for anybody else who’s going to have to go through this.”* (>50 and < 70, C7[ACT, Web]).

*“Just because I think the research is important, and trials like this are massively important … it was a no brainer for me to be involved in something that potentially could help in the future.”* (≤50, C4[SMS, Web]).

Some participants felt their role in participating was to provide feedback about their trial experience.

*“My role is just to tell you about my experiences based on the feedback I provided in the questionnaire so that you can…gather all that information…build up a picture for future…and the people who might be helped in the future…so tiny part of a big machine.”* (>50 and < 70, C8[no components]).

**Retention:**

**TDF – Motivation and Goals/TDF – Memory, Attention, and Decision Processes**

Several participants reported reduced motivation to complete questionnaires. Reasonings included the length of the questionnaires, repetitive questions, and frequency of the questionnaires.

*“It was time consuming … cause it takes about between 30-45 minutes and you have to really commit to doing it when you’re doing it, which means…you maybe don’t read the questions as thoroughly as you should and you just come with the first thing that comes to mind.”* (>50 and < 70, C2[SMS, IL]).

*“For me there was too many [questionnaires], and I thought some of them were repeating themselves. Maybe I didn’t read them properly.”* (≥70, C2[SMS, IL]).

A few participants reported forgetting to complete the last questionnaire.

*“I only finished the last questionnaire I think yesterday because of everything that was going on in my life like the operation and that, it totally went out of my mind.”* (≥70, C2[SMS, IL]).

In addition, two participants described the emotional demands of the questions as demotivating.

*“You gotta be in the right frame of mind to do them [questionnaires] because they can sometimes be a little bit like … I just don’t want to be reminded of that today … I might be going through a little bit of a … down dip and I think I put it off because I just thought…I don’t wanna rate how I’m feeling because I’m feeling a bit rubbish.”* (>50 and < 70, C1[SMS, IL, ACT, Web]).

In contrast, several participants reported feeling motivated to complete the questionnaires. This was attributed to the perceived acceptability of questionnaire length, having time to complete them, and finding the questions reassuring by reflecting shared experiences.

*“It was acceptable to me yes [length]…I had to go back to it when I had more time to do it but that was absolutely fine ….* *It makes that clear at the beginning of the study anyway, so it wasn’t as if … they said it was going to be 15 minutes and then it was 40, so it was fine.”* (>50 and < 70, C6[IL, Web]).

*“I found the options quite interesting because when you’ve had breast cancer its obviously quite a new experience…I thought well that must be what other people have … indicated … with symptoms and it made me feel that like I wasn’t stupid or I wasn’t going round the bend…some of the questions … that you ask I’m like yeah I definitely have that or I feel like that…I found it … a bit of an eye opener.”* (>50 and < 70, C8[no components]).

## Discussion

This qualitative process evaluation, nested in the ROSETA fractional factorial pilot O-RCT, demonstrated this novel experimental design was, overall, acceptable to participants. However, our findings highlight key areas where constructs of trial acceptability could be improved, such as coherence of randomisation procedures. Barriers and facilitators to recruitment and retention were similar to those reported in parallel group RCTs, indicating interventions addressing these issues could be applicable across all trials, regardless of designs.

In this pilot fractional factorial trial testing the feasibility of four behavioural intervention components to support medication adherence in women with breast cancer, overall, our findings suggest the complexity of the trial design did not substantially impact acceptability of trial experience. Coherence (understanding of the randomisation procedure) was the most mixed response among trial participants. However, this finding is not unique to factorial trials, as a substantial body of literature has demonstrated similar gaps in understanding of the randomisation process in parallel-group RCTs. ^34^ Unique to the design is that within factorial trials, participants have a higher probability of receiving at least one intervention component (e.g. 7/8 in the ROSETA pilot trial), compared with a parallel group RCT, where there is typically a 50:50 chance of receiving the intervention. Understanding is a key aspect of informed consent. ^35^ Therefore, studies within a trial (SWATs), exploring different approaches to explaining factorial trials and their benefits to participants, would be beneficial for future investigators conducting O-RCTs.

Findings related to retention primarily focused on the completion of questionnaires, which is expected given follow-up in the ROSETA pilot trial exclusively used self-report questionnaires, as opposed to visits or appointments. Comments on the length of questionnaire and repetition are not unique to this study or the factorial design used. ^36^ Balancing the need for data collection with participant burden should be considered for all trials during protocol development. However, additional consideration may be needed when using factorial trial designs, particularly if component-specific assessments are being used. For example, in the ROSETA pilot trial, participants were asked to complete an acceptability questionnaire for each intervention component they were randomised to receive. Participants randomised to receive all four components were therefore asked to complete an acceptability questionnaire four times, which understandably may have become repetitive and burdensome. ^25^ In some instances, this may be unavoidable, but there should be careful consideration as to the length of these assessments to minimise repetition and burden for participants, to facilitate retention.

Our exploration of recruitment in the ROSETA pilot trial indicated motivations to take part reflected those in other trials using different trial designs. For example, previous research investigating recruitment to RCTs has also identified several motivations for participation including personal benefit, ^36, 37^ altruism, ^34, 36^ and advancing science. ^34^ Overall, most barriers and facilitators to recruitment and retention identified in this study are not unique to trials using a factorial design. As such, it would be appropriate to draw on the wide body of literature of interventions to improve recruitment and retention to randomised controlled trials (for example telephone reminders, monetary rewards and prenotifications), that are likely to be applicable to trials using factorial designs. ^38, 39^

Our process evaluation has extended acceptability assessment beyond intervention components to trial experience. However, our study also has limitations. We did not include questions about all domains of the TFA and TDF to minimise participant burden, as participant interviews additionally focused on intervention component acceptability and fidelity. ^25, 26^ It is possible that other domains could have yielded further findings. Our deductive approach to analysis, based on the TFA and TDF, may have limited the depth of our analysis. The sample comprised only white women with breast cancer. Acceptability of trial experience, and barriers to recruitment and retention may differ in a more diverse sample. ^40^ We could not interview participants who withdrew from the trial or declined/did not respond to the interview invitation. This could have biased our findings, as only engaged, retained participants who potentially held more positive views about the trial were interviewed.

## Conclusion

Our qualitative process evaluation has demonstrated overall acceptability of participating in an optimisation trial using a fractional factorial experimental design. Our findings highlighted two areas of mixed acceptability: coherence of the randomisation process, and questionnaire burden. These are notable areas for improvement in future O-RCTs. Future SWATs could explore different approaches to explaining factorial trials to participants. Overall, many aspects of trial acceptability, recruitment and retention are not unique to factorial trial designs, indicating interventions addressing these issues may be applicable regardless of trial design used.

## Data Availability

The terms of the participant’s consent do not allow raw data to be made publicly available. The interview guides used are shared in the extended data. Open Science Framework: ROSETA Pilot Trial Process Evaluation https://doi.org/10.17605/OSF.IO/FP8ZW. ^29^ The project contains the following extended data:
• Participant Interview Guide • Blank Rapid Assessment Procedure Sheet Data are available under the terms of the Creative Commons Attribution 4.0 International license (CC-BY 4.0).
