# Supplementary material for: Exploring trial acceptability, recruitment, and retention in a pilot optimisation randomised controlled trial of a behavioural intervention to support medication adherence in women with breast cancer: A qualitative process evaluation
Source: NIHR Open Res. 2026 Jul 5;6:82. [Version 1] doi: 10.3310/nihropenres.14321.1 (PMC13562956; doi:10.3310/nihropenres.14321.1)
Supplement: Peer review history

# Reviewer report: Reviewer response for version 1

**DOI:** 10.3310/nihropenres.15604.r41517

**Published:** 2026-09-10

**Authors:** Brianna Leitzelar

**Affiliations:** 1 University of Minnesota-Twin Cities, Minneapolis, USA

Thank you for the opportunity to review your work!

**Summary**

 This manuscript describes the findings from a qualitative inquiry of trail feasibility and acceptability. Twenty individuals who participated in a 2 ^4-1 ^trial designed to test four intervention components to promote adherence to adjuvant endocrine therapy among women with breast cancer. Participants completed semi-structured interview following the 4-month assessment that focused on their perspectives of the intervention components and experiences within the research study. A rapid qualitative analysis using the specific theories and frameworks, and results were reported using illustrative quotes. Overall, the authors are to be commended for their work in conducting and writing up this work. This is an important topic and indexing this work contributes to advancing the field and supporting future similar research! I have a few comments for the authors to consider, which are outlined below by manuscript section.

 Intro

- The authors do an excellent job presenting their work clearly and accurately, using current literature to justify the importance of this work.
- I am curious to know more about why memory/forgetting, illness and medication beliefs, psychological flexibility, and living with side effects are the four intervention targets for the ROSETA program. Presumably this is in a previous publication related to the main trial, but it could be helpful to briefly summarize here as well.

Methods

- The use of the TFA and TDF frameworks to develop the interview guide is excellent.
- I’m curious as to why only 6 constructs were used for the TFA- could you include a brief rationale similar to that included for the selection of components for the TDF?
- **Participants:** The phrasing of the participants and recruitment suggests that the researchers were interested in recruiting a subset of participants, but in the description, it reads as though all individuals were invited and those who responded were interviewed. Could you clarify if there was a specific sample size target for the interview, or if everyone who was in the trial could participate.
- **Procedures**: The procedures are appropriate to the stated aims and study design. It is wonderful to see that a user advisory group contributed to question development! It could be helpful to include more information about the interviewers (e.g., number of interviewers, gender identity, cancer status, role in the trial), as this can contribute how the data was collected, how participants responses, and interpretations of the collected data.
- **Statistical Analysis**: The approach to analysis is clear and appropriate. I appreciate that the authors included the RAP sheets in supplemental material.

Results

- The results overall are clear and well-organized. I liked the illustrative quotes chosen.
- If available, it could be informative for readers to include some cancer- or treatment-related information (e.g., time since treatment, types of treatment- surgery, radiation, chemotherapy) in Table 4 (participant demographics), as their treatment information may influence their perceptions of the trial.
- The information in brackets after the illustrative quotes is a little unclear. To improve readability, I suggest the use of full words, rather than abbreviations, where possible. For example: (51-69 years old; Condition 3[text, leaflet]).

Discussion

- The discussion is excellently written and summarizes the key points within the current literature and addresses limitations of this work.
- I might add a few more strengths of this work to highlight the importance and value of using qualitative inquiry and grounding in theoretical frameworks to understand acceptability of trial participation.
- I may also suggest you add a limitation to describe how the interviewer perspective (e.g., being a member of the study team) could contribute to positive bias in the results.

Is the work clearly and accurately presented and does it cite the current literature?

Yes

If applicable, is the statistical analysis and its interpretation appropriate?

Yes

Are all the source data underlying the results available to ensure full reproducibility?

Yes

Is the study design appropriate and is the work technically sound?

Yes

Are the conclusions drawn adequately supported by the results?

Yes

Are sufficient details of methods and analysis provided to allow replication by others?

Yes

Reviewer Expertise:

mixed-methods research, behavioral research, cancer survivorship

I confirm that I have read this submission and believe that I have an appropriate level of expertise to confirm that it is of an acceptable scientific standard.

# Reviewer report: Reviewer response for version 1

**DOI:** 10.3310/nihropenres.15604.r41191

**Published:** 2026-09-08

**Authors:** Alex Molassiotis

**Affiliations:** 1 Aston University, Birmingham, England, UK

This is a well-written and timely qualitative process evaluation addressing an important methodological issue: participant experiences of an optimisation/factorial trial design. The study is theoretically informed and provides useful insights into trial acceptability, recruitment, and retention.

 If I would change anything (minor issues for me) I would suggest authors to:

 -Provide more detail on how the 20 interview participants were selected and how they compare with non-interviewed participants.

 -Explain why 20 interviews were considered sufficient and how sample adequacy was determined. Did you reach data saturation?

 -Expand the description of the rapid qualitative analysis process, including coding procedures, researcher involvement, and mapping to TFA/TDF constructs.

 -Participants interviewed had already agreed to participate and remain in the trial, which may have resulted in more positive views. This limitation should be discussed more explicitly.

 -The finding that some participants did not fully understand randomisation is important and warrants further discussion, particularly regarding informed consent and implications for future factorial trials.

 -Briefly explain optimisation RCTs and fractional factorial designs for readers unfamiliar with these methods.

 -State whether COREQ or other qualitative reporting guidelines were followed.

 -Consider including the interview guide as supplementary material.

 -Provide more detailed participant characteristics.

 -Expand discussion on questionnaire burden and implications for future studies.

 -Include a brief reflexivity statement.

Is the work clearly and accurately presented and does it cite the current literature?

Yes

If applicable, is the statistical analysis and its interpretation appropriate?

Not applicable

Are all the source data underlying the results available to ensure full reproducibility?

No source data required

Is the study design appropriate and is the work technically sound?

Yes

Are the conclusions drawn adequately supported by the results?

Yes

Are sufficient details of methods and analysis provided to allow replication by others?

Yes

Reviewer Expertise:

Supportive care; trial design in supportive care; randomised trials

I confirm that I have read this submission and believe that I have an appropriate level of expertise to confirm that it is of an acceptable scientific standard.
